# Labour migration of young women from the village to the capital in Bulgaria: A case study of woman servantry in Vakarel

**DOI:** 10.12688/openreseurope.16589.1

**Published:** 2024-01-10

**Authors:** Petko Hristov, Niya Spasova

**Affiliations:** 1 Institute of Ethnology and Folklore Studies with Ethnographic Museum, Bulgarian Academy of Sciences, Sofia, 1000, Bulgaria

**Keywords:** female labor mobility, servantry, Vakarel, Shopluk, social changes

## Abstract

The article is devoted to the maiden servantry in Bulgaria – a relatively late social phenomenon, but with important cultural and social consequences. It is also an indicator of the early modernization of Bulgarian society in the first half of the twentieth century. Using the example of Vakarel in Middle Western Bulgaria, the authors define servantry as a social mediator, facilitating the spread of urban culture and European models in the Shopluk villages around the capital Sofia. The emergence and development of female servantry as an enduring and sustainable social practice charts a path of change in marriage strategies and family patterns in a small rural community and thus in society as a whole. Gradually, from an escape from the adverse socio-economic factors and the harsh life in the village, temporary employment as a servant in the home of a wealthy family in the big city became an important moment in the young girl's growth and socialization, a condition for successful realization of family strategies in the rural environment. As a social phenomenon, servanthood has remained positively valued in the collective memory of the locals as part of the oral stories told within the family.

## Introduction

*'When you pass by the street wells in Sofia in the evening, have you noticed the maids gathering there to get water?*

*Look at them and you will discover some amazing things. You will discover the birth of a civilization that will later be spread all over the Sofia fields and mountains.*

**Figure 1.**
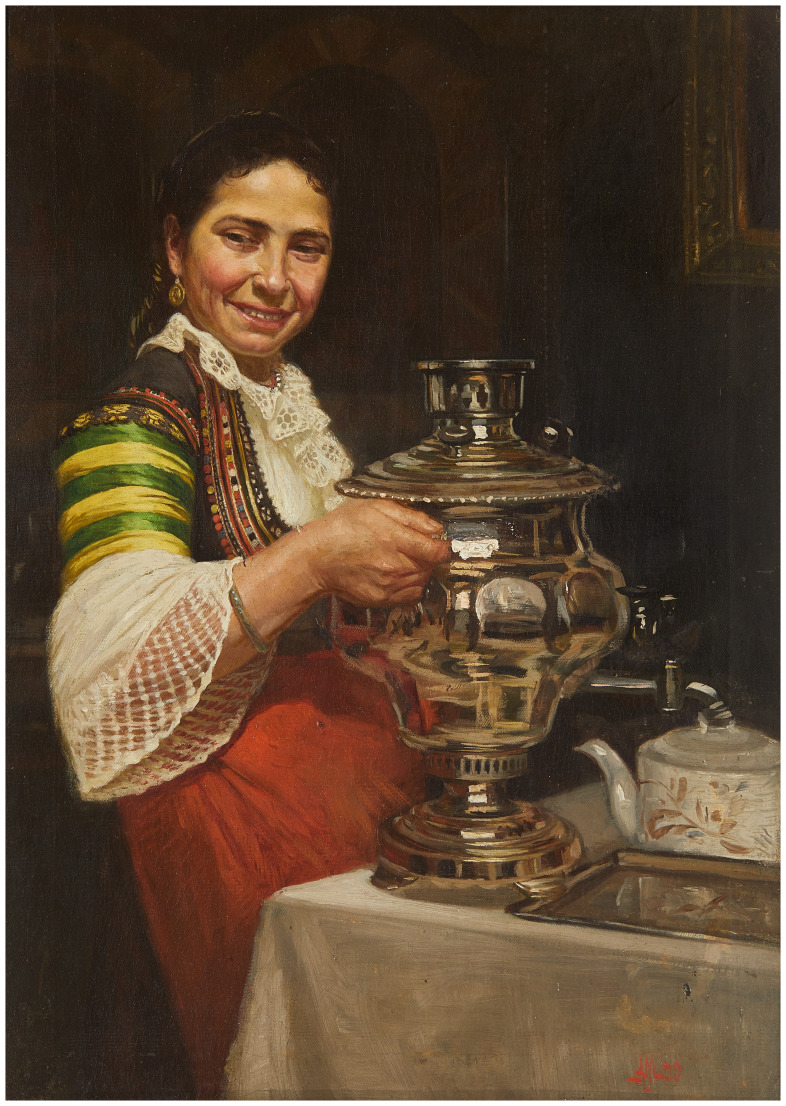
*Vakarel girl with a samovar* ( Anton Mitov, 1899). Sofia City Art Gallery, Inv. № 2319. This image is reproduced with the official permission of Sofia City Art Gallery.

*You will witness a spiritual process; you will see the changing of manners, language, costume, and notions, which have been left frozen by a whole chain of centuries and unstirred by any world-changing events*.' ^1^ ( Vazov, 1895) ( Figure 1)

**Figure 2.**
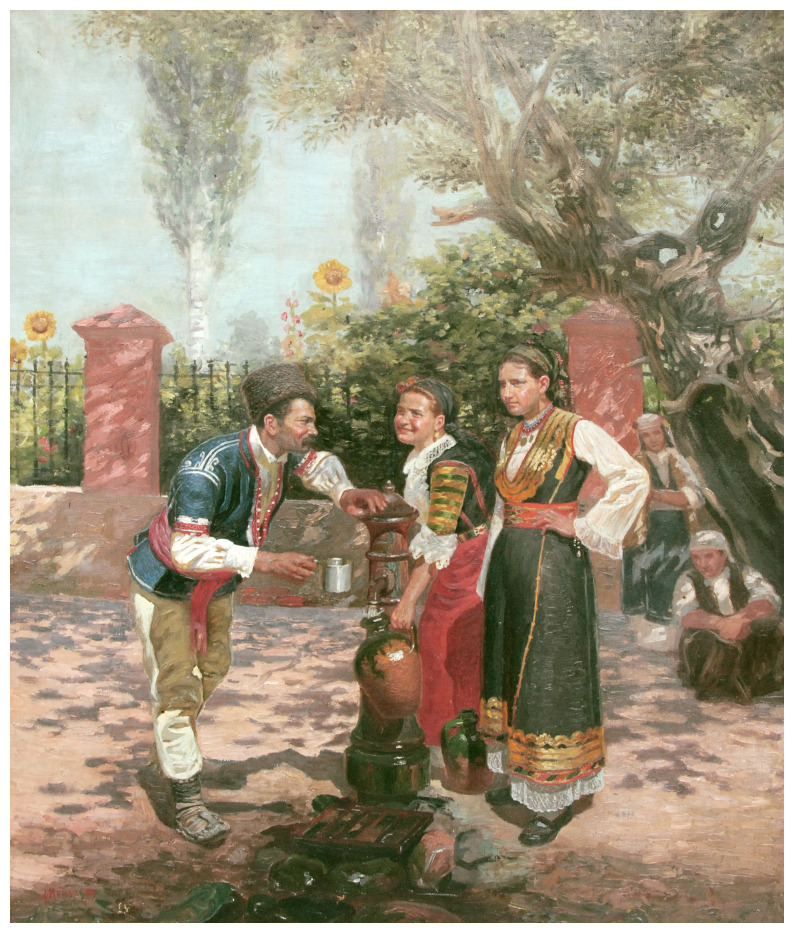
*At the fountain* ( Anton Mitov, 1899). Sofia City Art Gallery, Inv. № 2323. This image is reproduced with the official permission of Sofia City Art Gallery.

The words of Ivan Vazov, a classicist of Bulgarian literature, describe the social processes in the newly-proclaimed capital Sofia less than two decades after the establishment of the independent Bulgarian state, just liberated from the Ottoman Empire. Inspired by his observations of the capital's daily life, he published, in 1893 and 1895, a collection of short stories in two parts under the general title *'Draski i sharki'* ('Scribbles and Patterns'). In the second part, in the short story *'Who Will Civilize Shopluk'* he describes a servant girl from the nearby village of Vakarel as follows ( Figures 2).:

*'Look at that red-cheeked, beautiful Vakarel girl with her turned-up nose, pretty green headscarf, fine lace blouse, yellow-green sleeves of silk, and a red apron over a narrow, close-fitting tunic. The fine European lace and the slippers on her feet are the pleasant harbingers of the corset that will soon form part of her outfit. These beautiful Vakarel girls, not quite so wedded to prejudice and tradition, are the first to set the fashion trends in the servantry world of the capital city.'* ( Ivan Vazov, 1895).

The narrative continues with dialogue between the Vakarel maid and her admirer, a soldier from the capital.

Decades later, in his anthropogeographical description of Vakarel, Guncho Gunchev would write in his general description of the economic life of the Vakarel people: “The Vakarel economy also generated significant income through the maids who found well-paid jobs in our capital immediately after the Liberation” ( Gunchev, 1933, 141). He is also the author of the only study of the Vakarel servantry social phenomenon so far, carried out in 1930. In the period between 1920 and 1926, 70 % of the female population in Vakarel between the ages of 14 and 20 were employed, for various periods, in Sofia as maids ( Gunchev, 1941, 140). In that same year of 1930, according to this survey, 358 Vakarel girls were temporarily employed as maids almost entirely in the capital, with only six in other occupations: four seamstresses, one knitter, and one tobacco warehouse worker, from the various neighbourhoods (20 in total) of Vakarel ( Gunchev, 1941, 140–142). Although this number is not large in the general picture of servantry in Sofia ^2^, the Vakarel servants were becoming a trademark of the metropolitan labour market and were the highest-paid maids. It is no coincidence that, as early as 1880, passing through Vakarel, Konstantin Irechek would write in his travel notes: “The servants in Sofia are mostly from here” ( Irechek, 1899, 110). They were employed in the homes of doctors, apothecaries, merchants, military officials, and ministers ( Mitev, 2017, 345).

This article focuses on the social phenomenon of servant labour as one of the first forms of female labour mobility and an important channel for the penetration of urban cultural patterns in rural Bulgaria. The example of Vakarel is emblematic of the history of early modernization of the Bulgarian village in social and cultural aspects. The main research question of the field study we have done in Vakarel was whether the maids are the ones who change the lifestyle, the mores, the stereotypes of behaviour, and cultural patterns in the villages around Sofia. Our research is based on oral narratives (12 in total) and memories of the descendants of the maids from Vakarel and the nearby towns of Ihtiman and Belitsa ^3^. The beginning of the third millennium gives us the final opportunity to gather raw material for scientific analysis from women who practiced this occupation in the first half of the last century.

After WW II domestic servants became an object of investigation mainly for historians, who began to study them with new curiosity thanks to the spread of social history, the history of the family, historical demography, and (particularly from the 1970s) women’s and gender history ( Sarti, 2015, p. 32). In ethnology, late marriage and long servitude outside the native household are of important indicators of what historians and demographers call the ‘European Marriage Pattern’. In 1965 in historical demography a very influential essay by John Hajnal was published, who wrote that Western Europe was characterized by a peculiar marriage pattern with a high proportion of single people and marriages at a late age (cf. Hajnal, 1965, pp. 101–135). As Jack Goody emphasizes: ''‘European Marriage Patterns' were the subject of an article by Hajnal in which he draws a contrast between Western Europe and the totality of 'non-European', 'traditional', or 'developing' countries (including Eastern Europe)” ( Goody, 1983, p. 8). In contrast to the Eastern European and Mediterranean ‘Joint Family’ model, the presence in the sedentary household in Western Europe of not necessarily married individuals represents a considerably freer system of labour recruitment ( Mitterauer, 1986, p. 115). This opened up possibilities for the further development of different forms of labour organization, for a different attitude to women's wage labour, where maiden servitude provided new skills and urban patterns of domestic labour that she would later apply as a village housewife (ibid., p. 125). As Theresa McBride sums up in her landmark study ‘The Domestic Revolution. The Modernisation of Household Service in England and France 1820–1920’: “Almost paradoxically it [domestic service] served as a means of the modernization of rural labour and particularly of women,” during industrialization and urbanization ( McBride, 1976, р. 117).

Since the 1970s, many works have focused on domestic service as a crucial channel for both migration and social mobility (upwards and downwards), particularly for women ( Sarti, 2015, p. 35). Scholars like Peter Laslett and Michael Mitterauer identified domestic ‘service’ (as they called it) as part of the lifecycle in the typical West European demographical pattern. At first, young people worked for some years in the households of families other than their own, then – with the experience gained – married, established their households, and started (nuclear) families of their own. ( van Nederveen Meerkerk *et al.*, 2015, p. 9). Thus, servility and late marriage are inferred as characteristics only of so-called ‘European Marriage Patterns’. In ethnology, however, it has already been shown that the South Slavic *zadruga*, recognized as the ‘Joint Family’, is not only not a predominant pattern of family household ( Todorova, 2006, p. 207) but rather a desirable but rarely achieved pattern in the social structure of the Balkans ( Hristov, 2014, p. 6).

In our understanding, servantry is one form of domestic work ^4^, which both in the past and today is characterized by a great feminization ( van Nederveen Meerkerk *et al.*, 2015, p. 1). Although it has long attracted the attention of historical demographers, feminist labour history, and migration historians (cf. Sarti, 2015, p. 25), such a study is being done for the first time in Bulgarian social history and historical ethnology. Serfdom in Bulgaria between the two world wars was a form of internal migration that involved rural or small-town women seeking a wage income in an urban environment – “a common pattern all over the world, be it ancient India, preindustrial and industrializing Europe, or present-day Africa” ( van Nederveen Meerkerk *et al.*, 2015, p. 10). This study attempts to contextualize and concretize the picture of women's (specifically girls') labour mobility, which gained significant dimensions in the early twentieth century, in the societal structures and cultural practices of Bulgarian society in the interwar decades.

On the other hand, it would allow historical patterns of female labour mobility to be mapped out and compared with contemporary forms of feminized labour migration in the last decade of the twentieth century and the beginning of the new third millennium.

According to historians who recorded the oral history of Vakarel, young Vakarel women were employed as maids in Samokov as early as the first half of the nineteenth century ( Gunchev, 1941, pp. 134–135). Within the Ottoman Empire (before 1878), girls from the region served mostly in rich Turkish, but also in Bulgarian and Jewish, families in the town, which at that time was one of the most developed Bulgarian urban centers during the second half of the nineteeth century. Before 1878, Vakarel maids were rarely employed in Sofia, but there were cases of them even in Constantinople, as in the case of Stoyanka Hristova, called 'The Consul' ( Mitev, 2017, p. 344). The rapid expansion of the newly-proclaimed capital Sofia and the increasing needs of the new Bulgarian bourgeoisie, as well as the demographic explosion in the Vakarel neighbourhoods after the Liberation, led to a rapid growth of domestic service from Vakarel in the capital city. And despite the competition from young girls who came from the villages of Pirdop, Tran, Tsaribrod (today Dimitrovgrad in Serbia), and Radomir after WWI, the first half of the 20th century marked the dominance of Vakarel maids in the capital's labour market.

In many respects, servantry inherited the tradition of young women working during harvest periods ( Marinov, 1984, p. 271). Going 'to the field' was popular even until the 1940s among the mountain villages around Sofia, as the women came down *en masse* from the region of Ihtiman (including Vakarel) to the villages in the Sofia area as well as to the Pazardzhik region ( Mitev, 2017: 339–340). After WWI, this type of labor mobility gradually declined as agricultural machinery entered the villages ( Bliznashka, 1984, pp. 116–117). As in other mountainous regions of Bulgaria, such as Gabrovo, for example, in the villages around Sofia, there is a reorientation and change in girls' labour mobility (Cf. Hristov & Spasova, 2022, pp. 236–256). The rapid population growth in the capital and the concentration of wealthy families led to an increase in demand for domestic servants, preferably with already-acquired experience. In Sofia, as early as the end of the 19th century, the *Dyulgerska Piazza* – the place in the capital where one could hire construction workers for the accelerated construction of public buildings ( Hristov, 2005: 87), began to organize twice a year the so-called *Sluginski Pazar* (Servant's Market). For one week after *Gergyovden* (St. George's Day) in May and after *Dimitrovden* (St. Demetrius Day) in October at Trapezitsa Square, they gathered girls from the mountainous districts of Central Western Bulgaria (Tran, Tsaribrod, and Godech) and from the surrounding rural areas of Vakarel and Samokov, who are employed by rich families. Younger girls are brought to the Servant's Market by their parents (mostly mothers), girls from farther away are brought by special organizers (dragomans), and the already experienced maids, such as those from Vakarel, make their own arrangements with the ladies they will serve in the household. In addition to the opportunity for labour migration and social mobility ( Sarti, 2015, p. 35), the appearance of this type of employment has one more very important consequence – young girls who worked and earned their own money became more independent over time ( Palairet, 1987, p. 34).

According to the information gathered ^5^, hiring a servant from the region in question was mostly done through personal recommendations and acquaintances. A characteristic feature of the settlements around Sofia, such as those in the Ihtiman area, is the lack of a *dragomanin*, a very important middleman for the recruitment of servants from the more remote regions of the country ^6^. The need for such a figure disappeared due to the close geographical location and familiarity with the area. People living in the nearby settlements often went to the capital to sell their products and procure necessary household products and raw materials. Therefore, the big city did not seem so distant to them, and going there was easy. It was a normal routine for them to walk around the village square and establish contacts that would help them find a suitable family for their girl to work for. There are also accounts of girls finding a job themselves:

*'And the ones who went to the cities, they went to look after children. And there were a lot of them. Some went on their own, others were taken by their fathers. Most of them got married in Sofia. They were employed there permanently. Some of the girls said, 'My father took me there. I stayed for a while, but my master drank too much and would go out and leave me with the children.' And she went out in the street and met a man. She told him about her difficult situation and he took her to look after his two children. She found herself a good friend, a man, and she got married there'*

(Interviewee 1, b. 1928 in the village of Ochusha, Archives IEFSEM No. 1103 - III).

Proximity to Sofia also suggests an easier commute, especially from Vakarel by rail. It was therefore common for girls to spend the winter months, from *Dimitrovden* (or *Mitrovden*) in October to *Gergyovden* in May, working as servants, and return to the village in the summer to help with field work and harvesting. These girls' jobs in the city consisted mainly of taking care of children. It was during this period of their lives, that they learned how to 'run' a household and raise children.

*'I learned a lot working there. Washing windows... God, I would climb up to wash the windows and I had to look after a child at the same time! It was 6–7 years old. So there I was washing the windows, and the mistress was out. And I was washing the windows and watching that the child did not fall. At least the child listened to me. It called me 'kaka'* (bigger sister). *I slept in the kitchen. There was a special bench there, where you’d put your quilt, your mattress, and the sheet... Yes, a bench. My mother at home would sew, knit, and watch the children. I couldn't do all that there. How can you knit when you have to scrub the rooms every day? I only had Sundays off, when I used to look after my grandfather and sing songs to him. When he refused to eat, I had to slap him. I had to make him eat.'*

(Interviewee 2, b. 1927 in Vakarel, Archives IEFSEM No. 1103 - III).

The already-established reputation of the Vakarel girls as skilled and dexterous domestic servants enabled them to choose and set the conditions of their employment. One such dialogue at such a negotiation (' *tsanene*'), recorded by Hristo Brazitsov, was placed in the ‘Kragosvet’ magazine in November 1929:

*We stop in front of a girl of 15. She looks like an experienced servant. Her shoes look posh, and she has a bit of make-up on:*

- *'Are you free', we were about to say 'girl' and we stopped ourselves so as not to offend her, 'Miss?'*
- *'Course I'm free, why else would I be hanging around here?'*
- *'We're just being polite. What's your price?'*
- *'How many of you are there in your house?'*
- *'There are three of us: a man, a woman, and a child.'*
- *'I’m not working in a house with a child!'*
- *'Why not? The child’s as good as gold! And it's not little – five years old already.'*
- *'Yeah, right! The worst age! Not falling for that one!'*
- *'How about we give the child away so you can come and work for us?'*
- *'I never joke when I discuss business.*' (cf. Ivanova, 2020).

It is interesting to note that the servants were almost exclusively from Vakarel and some surrounding mountain villages. There are almost no records of girls from the nearby town of Ihtiman – “From Vakarel, many maids went to Sofia, but here, from Ihtiman, only a few” (Interviewee 4, b. 1928 in Ihtiman). The explanation, according to the locals, lies in the higher social status of the town. Due to its key geographical location, on the main road between Sofia and Plovdiv, continuing to Constantinople, Ihtiman was famous for being richer, and its inhabitants were mainly merchants and craftsmen. The respondents' accounts testify that Ihtiman was “rich enough that the girls did not have to work as maids, but poor enough that the locals could not afford to hire a maid” (ibid) ^7^.

The harsh socio-economic conditions in the country in the decades between the two world wars inevitably had an impact on the spread of the servantry phenomenon and the rapid increase in the number of young girls engaged in this activity ( Mitev, 2017, p. 346). However, this was not the only reason. The case of Vakarel, Ihtiman, and their nearby villages is also an outstanding example of a very important feature of this relatively new phenomenon. Very quickly, in just a few decades, this type of female labour mobility also gained widespread popularity because of the knowledge and skills that girls accumulated in the big city. And these, in turn, have become more highly valued over the years. The accounts of the respondents we had the opportunity to talk to testify unequivocally that, even in the earlier decades, in the late nineteenth and early twentieth centuries, the Vakarel women were much more open to the world and interacted with many different people passing through their villages. The knowledge and experience gained in the big city elevated the girls in the social hierarchy of the small local community and made them desirable daughters-in-law and wives. Many of them also learned a trade, especially tailoring and cookery, which was another reason for parents to take their girls to the city:

*'I'm very happy in Sofia and I didn't want to go to the village. I wanted to stay. You stay from Mitrovden to Gergiovden. My neighbor Mileta goes back to the village too often. And all the boys in the village laugh at her. If you have not been a servant in the city, you know nothing. You must do that. I didn't want to study, so I was sent to serve in the city. The girls would study for four years and then start work.'*

(Interviewee 2, b. 1927 in Vakarel, Archives IEFSEM No. 1103 - III).

In many ways, the social skills acquired in the city replace and even surpass the need for schooling, at least in the eyes of the local community in Vakarel.

**Figure 3.**
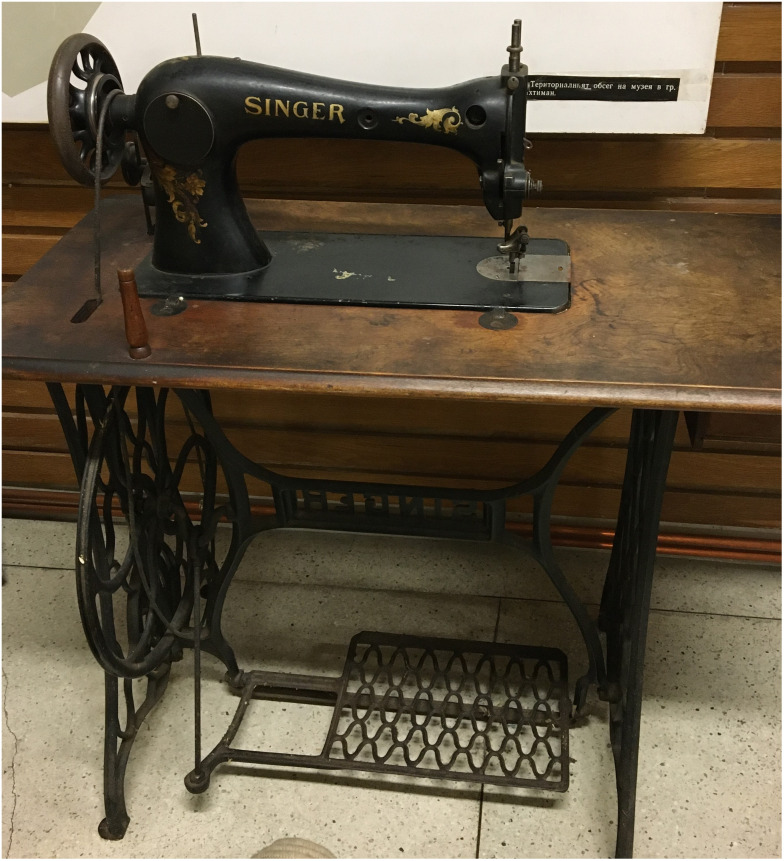
Singer sewing machine of Interviewee 2, b. 1927 in Vakarel (photo taken Petko Hristov, 2020).

In those first decades of the last century, the sewing machine, and especially the Singer machines, became a symbol of social status and skills ( Figure 3). By learning to sew, the young girl also mastered a craft that guaranteed her living income. This is also the observed mechanism by which new skills became increasingly valued. The sewing machine was a common gift from the owners to the girl when she left their house.

*'They go into servantry from the early age of 7–8. And they get their upbringing there. Some are taught to read and write, to embroider and sew. That's how they learned. A cousin of mine came back here to Ihtiman and became a seamstress. And it was very difficult to have your own sewing machine back then. It was expensive! The maids would give their wages to their parents – to feed the little children. After privatization, many came back to the village.'*

(Interviewee 1, b. 1928 in the village of Ochusha, Archives IEFSEM No. 1103 - III).

The nature of urban living is another reason for the growing popularity of this type of labor mobility among young girls. One reason for this was the free time they had on Sundays, something unknown to the rhythm of rural life. The new forms of entertainment appealed to girls, as did the freedom provided by the city.

*'Some girls got married in Sofia. That’s why we all promenaded along Regent Street – to meet boys and girls of our age! The party was there, in Poduene! My mistress took me there and introduced me to a good lad, but I didn't fancy Sofia. 'I’m going back to Vakarel, I am!' No idea why! That was how I saw the world back then! I'm thinking now about those times… My mistress wasn't a bad woman, you know. She couldn't wash her plate, but she was all right, she took me out with her. And here, here work never ends!'*

(Interviewee 2, b. 1927 in Vakarel, Archives IEFSEM No. 1103 - III).

**Figure 4.**
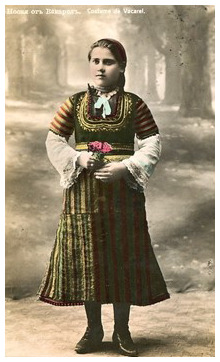
Postcard with a girl in Vakarel traditional costume. This image is reproduced from the official website of the Vakarel Municipality, which is freely accessible ( http://vakarel.eu/main_bg/11692_kartichki.html) (last accessed 30.08.2023).

In the context of urban life, it is important to note the clothing of the servants. It was in the city that young girls had the opportunity to change the way they dressed and began to express themselves through it ( Figure 4).

*'The maids would dress in a mixed fashion, half village and half city clothes. Their mistresses gave them city clothes.'*

(Interviewee 1, b. 1928 in the village of Ochusha, Archives IEFSEM No. 1103 - III).

*'We would dress up in our pleated skirts (fustanellas) when we went to the promenade. A fustanella, a jacket, and a headscarf. At a later stage, I started putting some lipstick on my lips and my cheeks. Earlier on, I didn’t know the first thing about anything! I would hurry to go back to the village, to see my girlfriends and the village… as if to make sure it was still there… I used to wear wooden or rubber slippers, but that stopped.'*

(Interviewee 2, b. 1927 in Vakarel, Archives IEFSEM No. 1103 - III).

Many of the respondents' accounts attest to the good treatment of the maids by the rich families. They not only provided food and shelter to the girls but also educated them, helped them to realize their life paths and secure a better future for themselves:

*'The masters treated my mother well, like their own child. They called her 'Rade' and she called them 'mistress' and 'master'. She hasn't told me much. But they were good to her, they bought her clothes. She arrived at theirs in her best village dress, but she bought city dresses, slippers, and shoes there… She was quite spoiled. She was the nanny of two little children there, and the mother was a dentist, I think, a very friendly woman. When there were no guests in the house, my mother ate with them, like a member of the family. When they entertained, my mother served them coffee and snacks. And I think she slept with the children, so they were not alone.'*

(Interviewee 3, b. 1927 in Ihtiman, Archives IEFSEM No. 1103 - III)

It is interesting to note that there was a positive attitude from the girl's parents as well towards the rich family:

*'But as far as I know, my Granny never stopped caring about the rich family. She felt she had to, as her child was with them. They* [in the village] *had eggs and cows… So Granny provided for them. And they liked each other, my Granny was a bubbly person.'*

(Interviewee 3, b. 1927 in Ihtiman, Archives IEFSEM No. 1103 - III)

There are, of course, accounts that speak of a not-so-favourable outcome in the fates of these girls:

*'When the little maids grew up, they would start socializing and promenading in the parks. Some of them had babies outside of marriage. Two girls from our neighbourhood did that. They left their children to the state. But one of them collected her child and brought it back to her parents in the village to look after it, and she got married in another village. And her son grew up and got married to a very nice girl, so he did all right in the end.'*

(Interviewee 1, b. 1928 in the village of Ochusha, Archives IEFSEM No. 1103 - III)

In support of this, Trendafil Mitev writes, quoting Georgena Rusinova's account, that girls were subjected to ruthless exploitation of their child labor in bourgeois families ( Mitev, 2017, pp. 347–349). However, we cannot disagree with Guncho Gunchev, who writes about the important “cultural role that servantry played in Vakarel” ( Gunchev 1941, p. 143), thanks to the rapid change that Vakarel's maids underwent in the more cultured urban environment. He concludes: “In our tours in Vakarel and its surroundings, we have repeatedly seen the good aspects that servantry brought to the life of this region” (ibid).

We can only agree with this observation. Servantry has remained positively valued in the collective memory of the locals as part of the stories related to the family. Detailed studies in different regions of the country are yet to be carried out and using an interdisciplinary approach in studying individual segments of the observed social process and cultural changes, comparing the different elements of it, and highlighting the commonalities and observed differences, is a successful scientific strategy, in our view.

From the perspective of the social history of Bulgaria and the early modernization of Bulgarian society, still agrarian in its entirety in the first half of the twentieth century, servantry is a relatively late phenomenon of female labour mobility, which very quickly gained mass popularity in the years after WWI and became a leading form of domestic labour. Statistical data from the first half of the twentieth century show that, while the total number of domestic servants in Bulgaria remained within a stable range, the number of men declined dramatically in the period between the two world wars, a trend that has persisted over the years, according to 'Active and Inactive Population at 31.XII.1926 by category, occupation, and status, in the occupation 'Unspecified trades and other' the following data are present:

Domestic work and domestic servants – 21,295 (0.4 %).
- Domestic servants and domestic work:
1910 - 26,407 in total; of these, 3,476 men;
1920 - 20,994 in total; of these, 2,191 men;
1926 – 21,295 in total; of these, 1,824 men ( Spasova, 2019, p. 5)

Thus, from its inception, servantry became extremely widespread throughout the country. Of course, the greatest percentage of girls went to the capital and the big towns, because of the concentration of wealthy families there who could afford to hire household help. Our research shows that the benefits of hiring a domestic servant were two-way: girls increased their qualifications and widened the perimeter of their marriage choices, while housewives themselves enjoyed the prestige of having a maid in their house. This fact elevated them in the hierarchy of the modernizing Bulgarian bourgeois society. This also explains the increasing popularity in the 1930s and 1940s of girls finding work in wealthy families through personal recommendations. In some cities, such as Ruse, for example, as early as the 1930s, agencies were opened where those wishing to be maids registered. In the capital city of Sofia, a kind of employment papers were introduced for long-servants, but recruitment continued to take place mainly at the twice-yearly Servant Market, as mentioned.

Servants were characters with a special place in the structure of both the Bulgarian city and the Bulgarian village during this period. In the countryside, they were the desired future wives who had mastered the urban model of life and household care. In the city, the maids were part of the mechanism for modernizing Bulgarian society, although they had not severed the threads of their dependence on agrarian rural culture. This type of dual social mobility is becoming an important part of the changing social structure of Bulgarian society and a form of modernization of the Bulgarian village.

The origin and development of female servantry as an enduring and sustainable practice is not only a mark of the early modernization of Bulgarian society. It also charts the path of change in family patterns and hence in society as a whole. Gradually, from an escape from adverse socio-economic factors and the harsh life in the countryside, temporary employment as a servant in the home of a wealthy family in the big city became an important moment in the young girl's growth and socialization: a condition for successful realization of family strategies in a rural environment. In many cases, the experiences they gained during their time as servants predetermined their choice of a marriage partner, and influenced their work habits, and the overall course of their lives. The knowledge and experience they acquired in the big city inevitably became part of their daily lives in the countryside as well. In this way, it becomes possible to trace the mechanisms and pathways through which new cultural patterns enter people's everyday lives. First, of course, it happened through clothing, food, and manners. Not only because they are most easily assimilated, but also because they are the key elements that distinguish social groups from one another. The desire to demonstrate a higher social status led to the need for self-improvement and inevitably to self-aggrandizement, part of which was the employment of a servant as part of the household. Because of all these factors, servantry remained widespread and popular for four decades, until the imposition of the new class ideology and Soviet-style socialist rule in Bulgaria after WWII. This gave rise to a different type of social change and demographic process, which is the subject of other studies.

## Data Availability

The data underlying the results of the interview transcripts cannot be made publicly available due to ethical restrictions to protect personal data of participants as sufficient deidentification of transcripts is not possible. The complete data is archived in the Ethnographic Archives of the Institute of Ethnology and Folklore Studies with Ethnographic Museum – cf. Archives IEFSEM No. 1103 – III [Address: Sofia 1000, Moskovska str. 6-D, on-site access]. To request access to the underlying data, readers must submit a declaration to the Director of the IEFEM stating the reason for use of archive materials via e-mail [ office@iefem.bas.bg] as outlined in the terms of use of the archive [ https://iefem.bas.bg/%d1%83%d1%81%d0%bb%d0%be%d0%b2%d0%b8%d1%8f-%d0%b7%d0%b0-%d0%bf%d0%be%d0%bb%d0%b7%d0%b2%d0%b0%d0%bd%d0%b5-%d0%bd%d0%b0-%d0%b0%d1%80%d1%85%d0%b8%d0%b2%d0%b8%d1%82%d0%b5.html].

## Footnotes

^1^ Vazov., Iv. [Koi shte civilizuva Shopsko?] Вазов, Ив. Кой ще цивилизува Шопско? – https://www.slovo.bg/old/vazov/draski/shopsko.htm (last seen 30. 08. 2023)

^2^ According to Guncho Gunchev, by 2 March 1930 in the capital, there were 18,022 maids registered in the Servantry Bureau. The Labour Inspectorate, however, did not categorize them as workers and, therefore, they could not benefit from the Public Insurance fund. (cf. Gunchev, 1941, 143).

^3^ The materials from the field survey are archived in the Ethnographic Archives of the Institute of Ethnology and Folklore Studies with Ethnographic Museum – cf. Archives IEFSEM No. 1103 – III [Address: Sofia 1000, Moskovska str. 6-A, on-site access] In Archives IEFSEM No. 1103 – III, it contains the full transcripts of the interviews with 12 women that the two authors conducted in 2020 year. Only some of the interviews are quoted in this article, indicating only the birth place and ages of the respondents. All of them are women and we have their verbal consent for their narratives to be quoted publicly in scholarly publications. The authors have full copyright to the archival data in Archives IEFSEM No. 1103 – III.

^4^ The authors of the collective monograph ‘Domestic Workers of the World: Histories of Domestic Work as Global Labor History’ define domestic work as “Work in the households of others includes all tasks concerning household work such as cooking, washing, cleaning as well as care work such as taking care of children, elderly and sick persons“ (cf. van Nederveen Meerkerk *et al.*, 2015, p. 2).

^5^ The data underlying the results of the interview transcripts cannot be made publicly available. See data availability statement.

^6^ About the region of *Tuzluk* in Northeast Bulgaria cf. Hristov & Spasova, 2020, pp. 210–225.

^7^ cf. Archives IEFSEM No. 1103 - III.
