# Supplementary material for: Labour migration of young women from the village to the capital in Bulgaria: A case study of woman servantry in Vakarel
Source: Open Res Eur. 2024 Jun 10;4:13. Originally published 2024 Jan 10. [Version 2] doi: 10.12688/openreseurope.16589.2 (PMC11195606; doi:10.12688/openreseurope.16589.2)
Supplement: Peer review history

# Reviewer report: Reviewer response for version 2

**DOI:** 10.21956/openreseurope.19430.r41358

**Published:** 2024-06-18

**Authors:** Hari KC

**Affiliations:** 1 Canada Excellence Research Chair (CERC) in Migration and Integration, Toronto Metropolitan University, Toronto, Ontario, Canada

I went through the response of the authors, and I would be happy to see this article indexed.

Is the argument persuasive and supported by appropriate evidence?

Partly

Is the work clearly and cogently presented?

Yes

Is the topic of the essay discussed accurately in the context of the current literature?

Yes

Does the essay contribute to the cultural, historical, social understanding of the field?

Yes

Reviewer Expertise:

My research is within the broader field of international migration and mobility, migration policy and governance, with a regional focus on Asia.

I confirm that I have read this submission and believe that I have an appropriate level of expertise to confirm that it is of an acceptable scientific standard.

# Reviewer report: Reviewer response for version 1

**DOI:** 10.21956/openreseurope.17906.r38733

**Published:** 2024-05-07

**Authors:** Otilia Hedesan

**Affiliations:** 1 West University of Timisoara, Timisoara, Romania

The article presents a very important aspect of Bulgarian society: the women migration for labour from the Bulgarian villages to the country capital, Sofia. The research follows the historical stages of this social process, using appropriate bibliographical references. 

 Studying the phenomenon of servantry in Sofia at the end of the 19th century and at the first half of the 20th century, the authors rightly note that this is "the first form of female labour migration" in Bulgaria. They also note the social effects of this migration for work (urbanization of rural life, transfer of knowledge and lifestyle from the city to village) and its importance in making different actual life stories.

Is the argument persuasive and supported by appropriate evidence?

Yes

Is the work clearly and cogently presented?

Yes

Is the topic of the essay discussed accurately in the context of the current literature?

Yes

Does the essay contribute to the cultural, historical, social understanding of the field?

Yes

Reviewer Expertise:

Cultural Anthropology, Ethnology

I confirm that I have read this submission and believe that I have an appropriate level of expertise to confirm that it is of an acceptable scientific standard.

# Reviewer report: Reviewer response for version 1

**DOI:** 10.21956/openreseurope.17906.r38731

**Published:** 2024-03-28

**Authors:** Hari KC

**Affiliations:** 1 Canada Excellence Research Chair (CERC) in Migration and Integration, Toronto Metropolitan University, Toronto, Ontario, Canada

**Comments**

 This is overall a strong paper where the authors use the concept of 'maiden servantry' from rural Bulgaria to its capital city at a specific historical moment and examine the way in which the phenomenon heralded the modernization processes of Bulgaria. Below are some comments and suggestions for the authors: 

 The authors aim to investigate “whether the maids are the ones who change the lifestyle, the mores, the stereotypes of behaviour, and cultural patterns in the villages around Sofia” and in order to do this, they have gathered oral narratives and memories of 12 research participants who are supposed to have been the descendants of the maids from Vakarel and the nearby towns of Ihtiman and Belitsa. How did the authors go about tracing the lineage between these research participants and those maids from Vakarel? From an epistemological point of view, how can the question that pertains to the past be answered using the data collected from the present? The authors in the first place would need to justify the choice of their methodology and address this epistemological incongruence between the main research question and the data. Secondly, the authors have not adequately engaged with the interview data they have collected and this means that there is a disconnect between the question and the evidence. The authors have made profuse use of archival and ethnohistorical data in the paper, but they have refrained from discussing this as a research method outrightly. The methodological rigour could be enhanced further by for instance combining the ethnohistorical and ethnographical methods.

 In the absence of a separate concluding section, the paper ends a bit abruptly. I advise the authors to add a concluding section. 

 The paper should be carefully edited for grammatical and technical errors, such as in citing page numbers, there is a lack of uniformity in the use of ‘p’ or ‘pp’ or no letters.   Also, the following paragraph needs proofreading for the proper use of tense: “Younger girls are brought to the Servant's Market by their parents (mostly mothers), girls from farther away are brought by special organizers (dragomans), and the already experienced maids, such as those from Vakarel, make their own arrangements with the ladies they will serve in the household.”

Is the argument persuasive and supported by appropriate evidence?

Partly

Is the work clearly and cogently presented?

Yes

Is the topic of the essay discussed accurately in the context of the current literature?

Yes

Does the essay contribute to the cultural, historical, social understanding of the field?

Yes

Reviewer Expertise:

My research is within the broader field of international migration and mobility, migration policy and governance, with a regional focus on Asia.

I confirm that I have read this submission and believe that I have an appropriate level of expertise to confirm that it is of an acceptable scientific standard, however I have significant reservations, as outlined above.

# Response

**Published:** 2024-04-17

**Authors:** Petko Hristov

**Affiliations:** Ethnographical Archives, Institute of Ethnology and Folklore Studies with Ethnographic Museum at BAS, Sofia, Bulgaria, Bulgaria

*Thanks for the helpful comments! The imposition of the socialist model in Bulgaria in the mid-1950s interrupted the practice of servantry, and this necessitated the historical-ethnographic reconstruction based on ethnographic narratives, memoirs, publications from the relevant period and, of course, the stories of the direct participants, as far as they can be found and collected in modern times.* It is true that we wrote at the beginning that " *Our research is based on oral narratives (12 in total) and memories of the descendants of the maids from Vakarel and the nearby towns of Ihtiman and Belitsa*", but in fact the text we cite three female respondents, two of whom were actually servants in Sofia as girls (respondents 1 and 2) and one is the daughter of a servant and reproduces her mother's stories (respondent 3). In our opinion, there is no epistemological discrepancy because it is about oral history and preserved family memories. The text will be edited for technical inconsistencies, but it has been carefully edited grammatically by a native English editor. We will also consider supplementing the conclusion. Thank you! Authors
